# Seroprevalence, infection, and personal protective equipment use among Colombian healthcare workers during the COVID-19 pandemic

**DOI:** 10.3389/fpubh.2023.1225037

**Published:** 2023-10-10

**Authors:** Edgar O. Beltrán, Stefania Martignon, Carolina Coronel-Ruiz, Myriam L. Velandia-Romero, Consuelo Romero-Sanchez, Viviana Avila, Jaime E. Castellanos

**Affiliations:** ^1^Universidad El Bosque, Research Department, UNICA—Caries Research Unit, Bogotá, Colombia; ^2^Universidad El Bosque, Vicerrectoría de Investigaciones, Grupo de Virología, Bogotá, Colombia; ^3^Universidad El Bosque, Cellular and Molecular Immunology Group, InmuBO, School of Dentistry, Bogotá, Colombia; ^4^Universidad Militar Nueva Granada, Clinical Immunology Group-Hospital Militar, School of Medicine, Bogotá, Colombia; ^5^Universidad Nacional de Colombia, Grupo de Investigaciones Básicas y Aplicadas en Odontología, Bogotá, Colombia

**Keywords:** SARS-CoV-2, COVID-19, infection, biosafety, prevalence, health personnel

## Abstract

**Introduction:**

Healthcare workers (HCWs) are at the forefront of the COVID-19 response and frequently come into close contact with patients and their virus-contaminated body fluids. Recent studies have identified differential risks of infection and the use of personal protective equipment (PPE) among HCWs. However, available data might be interpreted with caution because of differences in the national health systems, local implementation issues, and adherence limitations to guidelines. A comprehensive description of infection, exposure at work, and biosafety habits during the COVID-19 pandemic has not been conducted among the HCW groups in Latin American populations.

**Objective:**

To describe SARS-CoV-2 seroprevalence, infections, and extent of PPE use during the COVID-19 pandemic among HCWs at three different times, including dental practitioners (DP), nursing assistants (NA), physicians (P), and respiratory therapists (RT), from Bogotá, Colombia. Methods: After IRB approval, this cross-sectional study included 307 HCWs. Participants provided nasopharyngeal swabs and blood samples to detect viral RNA (RT-qPCR) and IgM/IgG anti-SARS-CoV-2 (ELFA-ELISA) at baseline (BL) and two follow-ups. Infection prevalence was defined as the number of positive-tested participants (RT-qPCR and/or IgM). Data on clinical status and biosafety habits were collected each time.

**Results:**

Differential infection prevalence was found among HCWs through the study timeline (BL: RT-qPCR = 2.6%, IgM = 1.6%; follow-up 1 (45 days after BL): RT-qPCR = 4.5%, IgM = 3.9%; follow-up 2 (60 days after BL): RT-qPCR = 3.58%, IgM = 1.3%. Dental practitioners showed a higher infection frequency in BL and follow-up 1. IgG-positive tested HCWs percentage progressively increased from BL to follow-ups among the whole sample while index values decreased. Limitations in N95 availability and a high perception of occupational risk were reported.

**Conclusion:**

A low prevalence of active SARS-CoV-2 infections among HCWs groups was found. Over time, there was an increase in participants showing IgG antibodies, although the levels of these antibodies in the blood decreased. Additionally, HCWs reported limitations in the availability of PPE as well as a variation in their safety practices.

## Introduction

Severe acute respiratory syndrome coronavirus 2 (SARS-CoV-2), the etiological agent of the coronavirus disease 2019 (COVID-19) pandemic, has infected more than 676,609,955 million people worldwide and led to 6,881,955 million deaths (1). Although SARS-CoV-2 infection has a lower mortality rate than those caused by other viruses belonging to its family (SARS or Middle East Respiratory Syndrome), it was able to trigger the pandemic owing to its spread through symptomatic and asymptomatic carriers (2). Asymptomatic carriers contributed substantially to the virus spread, even when breathing indoors (3).

The risks associated with SARS-CoV-2 infection or those caused by other respiratory viruses are not well understood among healthcare workers (HCWs) (4). HCWs refer to the staff in healthcare services whose job responsibilities require them to remain indoors, thus rendering them exposed to potentially infected patients (4). Additionally, close contact with such patients and other potentially infected staff members increases the possibility of transmission through splatters, droplets, or aerosols. These aspects of the HCWs' job responsibilities heighten the risk of infection and virus spread (5–7).

In Colombia, as in other Latin American countries, the national public health surveillance system made vital efforts to adapt health services to counteract the risks associated with the COVID-19 pandemic (8). However, social inequalities and poverty were exacerbated during the pandemic (9). In Colombia, this period was characterized by the low availability of resources for healthcare, a low number of hospital beds (less than 2.1 per 1,000 habitants), lack of comprehensive care for patients, violence against human rights defenders, mass social protests, tax reforms that imposed new fiscal obligations on the middle socioeconomic class, and increased taxes on basic living needs (9).

Several studies have reported that HCWs on the frontlines in various countries may have higher rates of SARS-CoV-2 infections than that reported, suggesting that the actual number of cases among Colombian HCWs could be underestimated (10, 11). In addition, healthcare professionals have reported limited access to personal protective equipment (PPE) (12). Further, in Colombia, due to limited vaccine availability and access, the national vaccination plan against SARS-CoV-2 was initiated in two phases and five stages, beginning in February 2021 (13). HCWs providing care to confirmed COVID-19 patients were included in the first stage of the first phase, whereas the remaining HCWs were included in the second stage of the first phase. Further, the genomic analysis identified 52 circulating SARS-CoV-2 lineages in Colombia from 774 genomes and 19 polymorphisms (14).

Collectively, these facts highlight the importance of characterizing the risk of viral infections among HCWs. As this issue has not been reported in the Colombian context, this study aimed to describe, at three different times, the extent of PPE use during the COVID-19 pandemic, SARS-CoV-2 seroprevalence, infections, and SARS-CoV-2 exposure factors in a sample of HCWs from Bogotá, Colombia, including dental practitioners (DP), nursing assistants (NA), physicians (P), and respiratory therapists (RT).

## Methods

### Ethical approval

Ethical approval was obtained from the Institutional Ethics Committees of Universidad El Bosque (Resolution 013, 2020) and Hospital Militar Nueva Granada (Resolution 087, 2021). These covered the recruitment of HCWs from eight institutions (seven primary-care provider centers and one dental school). The HCWs voluntarily agreed to participate by providing signed informed consent. Data and samples were collected on-site from April to October 2021 (12–44th epidemiological week) under the current best clinical practices and protocols established by the Colombian National Government and the Consensus of the Colombian Infectious Diseases Association.

### Sample size and selection of participants

Sample sizes were calculated using the Sample Size ^®^ program version 1.1, based on the results of a similar study carried out by Chiu et al. where the prevalence of sick health workers with influenza was 21.6% (15). To calculate the sample size, the precision formula was considered desirable in absolute units, with a type I error of 0.05 and a distance to the population proportion of 5%. Based on the above data, a minimum sample size of 261 subjects (65 in each group) was determined, including 20% additional for dropouts during follow-ups, for a total calculated sample of 313 HCWs. The seven primary-health-service hospital providers (*n* = 7) and the dental school clinic (*n* = 1) selected corresponded to institutions with the highest number of HCWs regarding the groups considered in this study. Sites were selected based on their location along Bogota (north, south, and central localities), intending to collect representative information from the whole city. The HCW groups, consisting of DP, NA, P, and RT, were selected based on their close contact (within 1 m) with the patients, an inherent characteristic of their research. This aspect has been reported as the main determinant of viral exposure through droplets/aerosols indoors in primary care services during the study's conduct (5). The inclusion criteria were as follows: current employment in a healthcare facility, willingness to provide nasopharyngeal swabs and blood samples (10 mL) at three different times—baseline (BL), after 45 days (follow-up 1), and after 60 days (follow-up 2), and completion of a related self-reported questionnaire at the three times mentioned above.

### Participants' data collection

This study was conducted from April to October 2021 (epidemiological weeks: 12th-44th). A second wave of infections occurred in the country from the 3rd to the 19th of June. As participants arrived at the meeting point at each institution, risk and health status self-reported questionnaires in paper format were included and adapted from previously validated ones, aiming to understand symptoms and the general global effects of the pandemic on dental practice (16–18). In addition, adherence to biosafety-related questions involved those included in the Ministry of Health and Social Protection guidelines at the time of conducting the study (19). All data were collected through a self-reported 17-item questionnaire at BL, follow-up 1, and follow-up 2, right before sample collection. Medical status data were collected at BL using a health record form.

In the questionnaires, the variables measured included sociodemographics, viral exposure at work, and adherence to biosafety measurements in practice (PPE use, general work habits, and level of viral exposure associated with outdoor practices). During the period of conducting this study, most of the HCWs had begun to restart their in-office work. For this reason, outside exposure to the virus was restricted to asking about social meetings, attendance in crowded indoor spaces, or tourism.

### Nasopharyngeal swab sample collection

Prior to nasopharyngeal swab sample collection, the professionals underwent surgical hand washing under aseptic conditions and thereafter donned protection barriers (coats, gloves, N95 respirators, and eye protection glasses) to guarantee biosafety and avoid contact with the participants and surrounding areas that could increase the risk of contamination.

The participant's head was tilted backward and immobilized. A sterile swab was inserted through the nostril into the posterior region of the nasal cavity. The swab was rotated for 10 sec to collect as many epithelial cells as possible and then gently pulled out. Individual samples were subsequently stored in a viral transport medium to preserve the quality of the sample and thereafter maintained under refrigerated conditions at 4°C until further use (20). Subsequently, following transfer to a containment laboratory, viral inactivation of the samples was carried out by the addition of an in-house lysis buffer followed by vigorous mixing using vortex agitation in a class II biological safety cabinet. All instruments used, such as vortex and micropipettes, were exclusive to this procedure.

### SARS-CoV-2 genome detection

Viral genome detection was performed by RT-qPCR using RNA purified from fresh nasopharyngeal swab samples as templates. A reaction mix containing buffer, dNTPs, magnesium, and enzymes (reverse transcriptase and polymerase) was used. Specific primers and probes were used to identify the SARS-CoV-2 nucleocapsid and envelope genomes. Human RNA was assessed in parallel for quality control purposes. Primers and probes used corresponded to those previously reported by the Centers for Disease Control and Prevention and Corman et al. (21). Based on these references, the protocol used was standardized previously in our lab. The CFX96^TM^ manager software was used according to the following protocol: reverse transcription: 55°C, 15 min; initial denaturation: 95°C, 3 min; amplification cycles: 95°C, 15 s (42 cycles), and final extension: 58°C, 45 s. Samples were analyzed using the CFX96 Biorad equipment.

The reported detection limit for SARS-CoV-2 is 3.9 copies per reaction. No cross-reactions with other coronaviruses or respiratory viruses were detected. A sample was considered positive if the cycle threshold (Ct) value was between 20 and 35 with the human internal control Ct of < 35. Additionally, for samples with Ct values between 36 and 40, only those with fluorescence >400 RFUs were considered positive. Negative samples were defined as those without a Ct value.

### Blood sample collection

Blood samples were collected by qualified laboratory analysts using biosafety protection barriers as described above and following protocols and recommendations based on the guidelines of the World Health Organization, the District Health Secretary, and the Colombian National Institute of Health.

The most appropriate vein of each participant was chosen for puncturing, and a tourniquet was placed above the selected site. The collection tube caps were cleaned with 70% alcohol prior to puncturing. Peripheral blood (10 mL) was extracted from the vein of each participant using sterile syringes and collected in BD Vacutainers—blood collection tubes (yellow caps). Aseptic and antiseptic protocols were followed at all times. Samples were refrigerated at 80°C until further processing.

### Detection of SARS-CoV-2 igm and igg antibodies

SARS-CoV-2 antibodies were detected in the serum of participants using an automated assay, the VIDAS^®^ SARS-CoV-2 IgM/IgG kit, following the manufacturer's instructions. The assay is based on the enzyme-linked fluorescent assay (ELFA) technique intended for qualitatively detecting IgM/IgG antibodies to SARS-CoV-2 in human serum. The presence of IgM antibodies is intended to identify individuals with recent exposure to SARS-CoV-2, whereas the presence of IgG antibodies is intended to identify individuals with an adaptive immune response to SARS-CoV-2, which indicates prior infection or vaccine exposure. The assay principle combines a two-step sandwich enzyme immunoassay method with a final fluorescence detection (ELFA) step. Briefly, serum aliquots obtained from centrifuged samples were diluted and incubated with recombinant SARS-CoV-2 antigen coated onto the interior of a single-use solid-phase receptacle (SPR) device wall. Thereafter, the presence of IgG in samples was specifically detected using anti-human IgG labeled with alkaline phosphatase. Finally, the substrate (4-methyl-umbelliferyl phosphate) was cycled inside and outside the SPR device. If the antibody was present, the conjugate enzyme catalyzed the hydrolysis of the substrate into a fluorescent product (4-methyl-umbelliferone). The fluorescence was measured at 450 nm. A relative fluorescence value (RFV) was generated by subtracting the background value from the final fluorescence value. The assays were performed on a standard (S1), a negative control (C2), and a positive control (C1) that contained a humanized recombinant anti-SARS-CoV-2 antibody, either IgM or IgG, depending on the assay. The instrument automatically calculated the results according to the S1 standard and generated an index value (i) (where i = RFV sample/RFV S1). The test was interpreted as negative when i was < 1.00 and as positive when i was > 1.00.

### SARS-CoV-2 infection case definition

At each time point, infection prevalence was assessed by the number of identified active infections as new or pre-existing based on RT-qPCR and/or IgM-positive tested samples (22). In addition, self-reported data regarding the presence of suggestive symptoms of respiratory infections in the last 14 days (fever, cough, respiratory distress, and/or respiratory difficulty) were also used to identify symptomatic infections. Laboratory results and clinical data were included in the algorithm to determine active SARS-CoV-2 infections. However, past infection or previous exposure to the virus SARS-CoV-2 or vaccines were determined by positive-tested IgG participants.

### Statistical analysis

Categorical data are presented as frequencies and percentages. The Shapiro–Wilk test was applied to identify the normality of the data distribution. Quantitative variables were presented as the number of observations in each item (percentage) and the median (interquartile range) if they did not have a normal distribution. Qualitative variables are described using proportions. To compare the difference between groups (i.e., profession), a one-way ANOVA was utilized for continuous variables with a normal distribution, and the Kruskal–Wallis test was used for non-parametric data. Qualitative variables were analyzed using Pearson's chi-squared test when the expected square values were ≥5. Otherwise, Fisher's exact test was used. Sociodemographic, clinical information, habits, and laboratory results from baseline, follow-up1, and follow-up 2 were summarized. All *p*-values were two-sided, and a *p*-value of <0.05 was considered to indicate significance. Statistical analyses were performed using GraphPad Prism 9.0 for Windows.

## Results

### Sociodemographic

A total of 307 HCWs were included in the final analysis. The median age of the participating HCWs was 36 years (range: 29–47 years) (Table 1). The distribution of HCWs by occupation was as follows: DPs (*n* = 86, 28.0%), NAs (*n* = 78, 25.4%), Ps (*n* = 76, 24.7%), and RTs (*n* = 67, 21.8%) (*p* < 0.001; Fisher's exact test). A total of 71 HCWs (23.1%) reported no current disease or medical treatment (Table 1).

**Table 1:** HCWs' sociodemographic.

**Variable**		**Level**	**Dental practitioners (DP)**	**Nursing assistants (NA)**	**Physicians (P)**	**Respiratory therapists (RT)**	**Total**	***p***
Participants		n (%)	86 (28.0)	78 (25.4)	76 (24.8)	67 (21.8)	307 (100)	*0.0195*ꬸ
Age (Years)		Median (IR)	35 (29–47)	35 (27–42)	36.5 (28–46)	46 (30–49)	36 (29–47)	
Gender *n* (%)		Female	65 (75.6)	69 (88.5)	49 (64.5)	60 (89.6)	243 (79.2)	* < 0.001*ꬹ
		Male	21 (24.4)	9 (11.5)	27 (35.5)	7 (10.4)	64 (20.8)	
Comorbidity *n* (%)		No	68 (79.1)	61 (78.2)	52 (68.4)	55 (82.1)	236 (76.9)	*0.222*
		Yes	18 (20.9)	17 (21.8)	24 (31.6)	12 (17.9)	71 (23.1)	
Working hours per week		Median (IR)	40 (37–44)	48 (46–60)	48 (37–60)	48 (45–48)	48 (40–50)	0.0001ꬸ
Work activities stop (days)		Median (IR)	14.5 (14–20)	15 (14–30)	14 (7–40)	14 (10–15)	14.5 (10–21)	*0.2034ꬸ*
Stop working activities	BL	No	68 (79.1)	64 (82.0)	57 (75.0)	51 (76.1)	240 (78.2)	*0.719*
		Yes	18 (20.9)	14 (18.0)	19 (25.0)	16 (23.9)	67 (21.8)	
	Follow-up 1	No	78 (90.7)	68 (87.2)	70 (92.1)	60 (89.6)	276 (89.9)	*0.774*
		Yes	8 (9.3)	10 (12.8)	6 (7.9)	7 (10.4)	31 (10.1)	
	Follow-up 2	No	81 (94.2)	78 (100)	76 (100)	66 (98.5)	301 (98.0)	*0.017*ꭝ
		Yes	5 (5.8)	0 (0.0)	0 (0.0)	1 (1.5)	6 (2%)	
Travel 14 days before data collection *n* (%)		No	83 (96.5)	77 (98.7)	72 (94.7)	66 (98.5)	298 (97.1)	0.470
		Yes	3 (3.5)	1 (1.3)	4 (5.3)	1 (1.5)	9 (2.9)	
Social activities attendance	BL	No	76 (88.4)	76 (97.4)	70 (92.1)	67 (100)	289 (94.1)	0.005ꭝ
		Yes	10 (11.6)	2 (2.6)	6 (7.9)	0 (0.0)	18 (5.9)	
	Follow-up 1	No	86 (100)	74 (94.9)	74 (97.4)	61 (91.0)	295 (96.1)	0.018ꭝ
		Yes	0 (0.0)	4 (5.1)	2 (2.6)	6 (9.0)	12 (2.9)	
	Follow-up 2	No	86 (100)	78 (100)	76 (100)	67 (100)	307 (100)	Not calculated
		Yes	0 (0.0)	0 (0.0)	0 (0.0)	0 (0.0)	0 (0.9)	
Exposure (outside work)	BL	No	69 (80.2)	72 (92.3)	62 (81.6)	64 (95.5)	267 (87.0)	0.009ꬹ
		Yes	17 (19.8)	6 (7.7)	14 (18.4)	3 (4.5)	40 (13.0)	
	Follow-up 1	No	84 (97.7)	73 (93.6)	73 (96.0)	57 (85.1)	287 (93.5)	0.019ꭝ
		Yes	2 (2.3)	5 (6.4)	3 (4.0)	10 (14.9)	20 (6.5)	
	Follow-up 2	No	86 (100)	78 (100)	76 (100)	67 (100)	307 (100)	Not calculated
		Yes	0 (0.0)	0 (0.0)	0 (0.0)	0 (0.0)	0 (0.9)	
		Yes	5 (5.8)	0 (0.0)	0 (0.0)	1 (1.5)	6 (2%)	

Main characteristics collected through survey/clinical health records in the whole study or at each time point. P-values were calculated using the chi-squared (ꬹ), Fisher exact (ꭝ), or Kruskal–Wallis (ꬸ) tests. Not calculated: No comparisons were made when extreme data were compared.

### PPE use and biosafety

At BL, 254 HCWs (82.7%) reported being supplied with PPEs by their institution, and 42 (39.8%) were informed of the general restrictions, limited access, or instructions regarding the use of PPE. Further, most HCWs (93.7%) reported using gloves, coats, and gowns, only coats (94.7%), and face shields (92.7%) during patient care. Furthermore, most HCWs (85.53%) reported using N95 respirators, whereas a few HCWs (5.8%) reported being provided with only one N95 respirator per week (Table 2). At follow-up 1, 4.6% of HCWs reported restrictions in the use of or access to PPE, whereas 14.5% reported not being supplied with N95 respirators by their institution and having to procure them themselves. Approximately one-fifth (21.8%) admitted to replacing their respirators weekly. At follow-up 2, the same number of participants reported using N95 respirators as in follow-up 1. However, a relevant number of HCWs (*n* = 52; 17.6%) reported replacing their respirators weekly. Surprisingly, 4.7, 31.6, and 11.5% of HCWs reported no adherence to COVID-19 guidelines at BL, follow-up 1, and follow-up 2, respectively.

**Table 2:** Use of PPE among HCWs during the study.

**Equipment**		**Baseline**					**Follow-up 1**					**Follow-up 2**				
		**Dental practitioners**	**Nurseassistant**	**Physicians**	**Respiratory Therapist**	***p***	**Dental practitioners**	**Nurseassistant**	**Physicians**	**Respiratory Therapist**	***p***	**Dental practitioners**	**Nurseassistant**	**Physicians**	**Respiratory Therapist**	***p***
Personal Protective Equipment (PPE) *n* (%)	0	1 (1.16)	3 (3.85)	0 (0.00)	0 (0.0)	*<0.001*ꭝ	1 (1.16)	3 (3.85)	0 (0.0)	0 (0.0)	*<0.001*ꭝ	0 (0.00)	2 (2.60)	1 (1.33)	2 (2.99)	*0.050*ꭝ
	1	83 (96.51)	60 (76.92)	51 (67.11)	60 (89.55)		83 (96.51)	60 (76.92)	51 (67.11)	60 (89.55)		73 (84.88)	73 (94.81)	66 (88.0)	60 (89.55)	
	2 or more	2 (2.33)	15 (19.23)	25 (32.89)	7 (10.45)		2 (2.33)	15 (19.23)	25 (32.89)	7 (10.45)		13 (15.12)	2 (2.60)	8 (10.67)	5 (7.46)	
Face Mask N95 *n* (%)	No	2 (2.33)	17 (21.79)	18 (24.66)	7 (10.45)	*<0.001*ꭝ	2 (2.33)	17 (21.79)	18 (24.66)	7 (10.45)	*<0.001*ꭝ	2 (2.33)	17 (21.79)	18 (24.66)	7 (10.45)	*<0.001*ꭝ
	Yes	84 (97.67)	61 (78.21)	55 (75.34)	60 (89.55)		84 (99.67)	61 (78.21)	55 (75.34)	60 (89.55)		84 (97.67)	61 (78.21)	55 (75.34)	60 (89.55)	
Disposable Coat *n* (%)	No	2 (2.33)	8 (10.26)	5 (6.85)	1 (1.49)	*0.061*ꭝ	2 (2.33)	8 (10.26)	5 (6.85)	1 (1.49)	*0.061*ꭝ	2 (2.33)	8 (10.26)	5 (6.85)	1 (1.49)	*0.061*ꭝ
	Yes	84 (97.67)	70 (89.74)	68 (93.15)	66 (98.51)		84 (97.67)	70 (89.74)	68 (93.15)	66 (98.51)		84 (97.67)	70 (89.74)	68 (93.15)	66 (98.51)	
Surgical Cap *n* (%)	No	1 (1.16)	8 (10.26)	8 (10.96)	2 (2.99)	*0.013*ꭝ	1 (1.16)	8 (10.26)	8 (10.96)	2 (2.99)	*0.013*ꭝ	1 (1.16)	8 (10.26)	8 (10.96)	2 (2.99)	*0.013*ꭝ
	Yes	85 (98.84)	70 (89.74)	65 (89.04)	65 (97.01)		85 (98.84)	70 (89.74)	65 (89.04)	65 (97.01)		85 (98.84)	70 (89.74)	65 (89.04)	65 (97.01)	
Gloves *n* (%)	No	1 (1.16)	7 (8.97)	7 (9.59)	4 (5.97)	*0.063*ꭝ	1 (1.16)	7 (8.97)	7 (9.59)	4 (5.97)	*0.063*ꭝ	1 (1.16)	7 (8.97)	7 (9.59)	4 (5.97)	*0.063*ꭝ
	Yes	85 (98.84)	71 (91.03)	66 (90.41)	63 (94.03)		85 (98.84)	71 (91.03)	66 (90.41)	63 (94.03)		85 (98.84)	71 (91.03)	66 (90.41)	63 (94.03)	
Face-shield *n* (%)	No	2 (2.33)	8 (10.26)	8 (12.33)	3 (4.48)	*0.045*ꭝ	2 (2.33)	8 (10.26)	9 (12.33)	3 (4.48)	*0.045*ꭝ	2 (2.33)	8 (10.26)	9 (12.33)	3 (4.48)	*0.045*ꭝ
	Yes	84 (97.67)	70 (89.74)	64 (87.67)	64 (21.05)		84 (97.67)	70 (89.74)	64 (87.67)	64 (95.52)		84 (97.67)	70 (89.74)	64 (87.67)	64 (95.52)	

P-values were calculated through the Fisher exact test (ꭝ).

### Serologic status and SARS-CoV-2 infections

**Figure 1:**
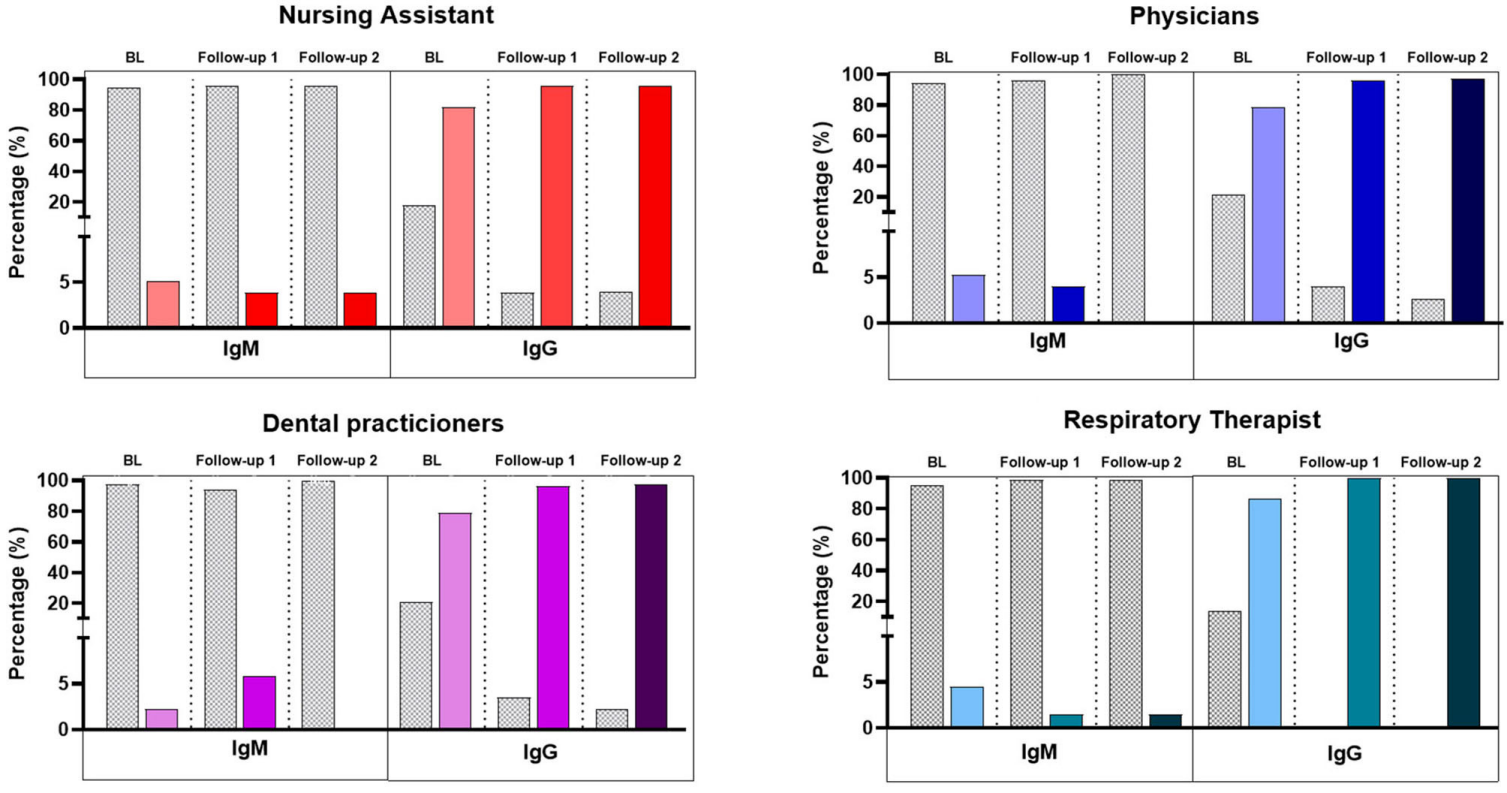
IgM and IgG against SARS-CoV-2 in HCWs. Percentages were calculated based on the total sample. Bars show IgM/IgG antibodies among the four groups of workers included in the study during the three times assessed.

Of the 307 HCWs whose serum samples were assessed for the presence of IgG and IgM antibodies by ELFA, 81.2% tested IgG-positive at BL, 97% at follow-up 1, and 97.7% at follow-up 2 (Figure 1). These data are consistent with the increase in the number of vaccinated HCWs; at BL, most HCWs (93.5%) had received at least one vaccine dose, and by the end of follow-up 2, most HCWs (96.4%) had received both doses of the 2-dose vaccination scheme. HCWs participating in the study had previously received vaccines manufactured by Pfizer (BioNTech), Sinovac (*CoronaVac*), Moderna, or AstraZeneca (*Vaxzevria*). The booster dose of the SARS-CoV-2 vaccine had not yet been approved until the end of the study period. Surprisingly, IgG index values decreased between the HCWs during the timeline (BL: 360.5±299.6; Follow-up2: 323.7 ± 228.6; p < 0.05).

**Figure 2:**
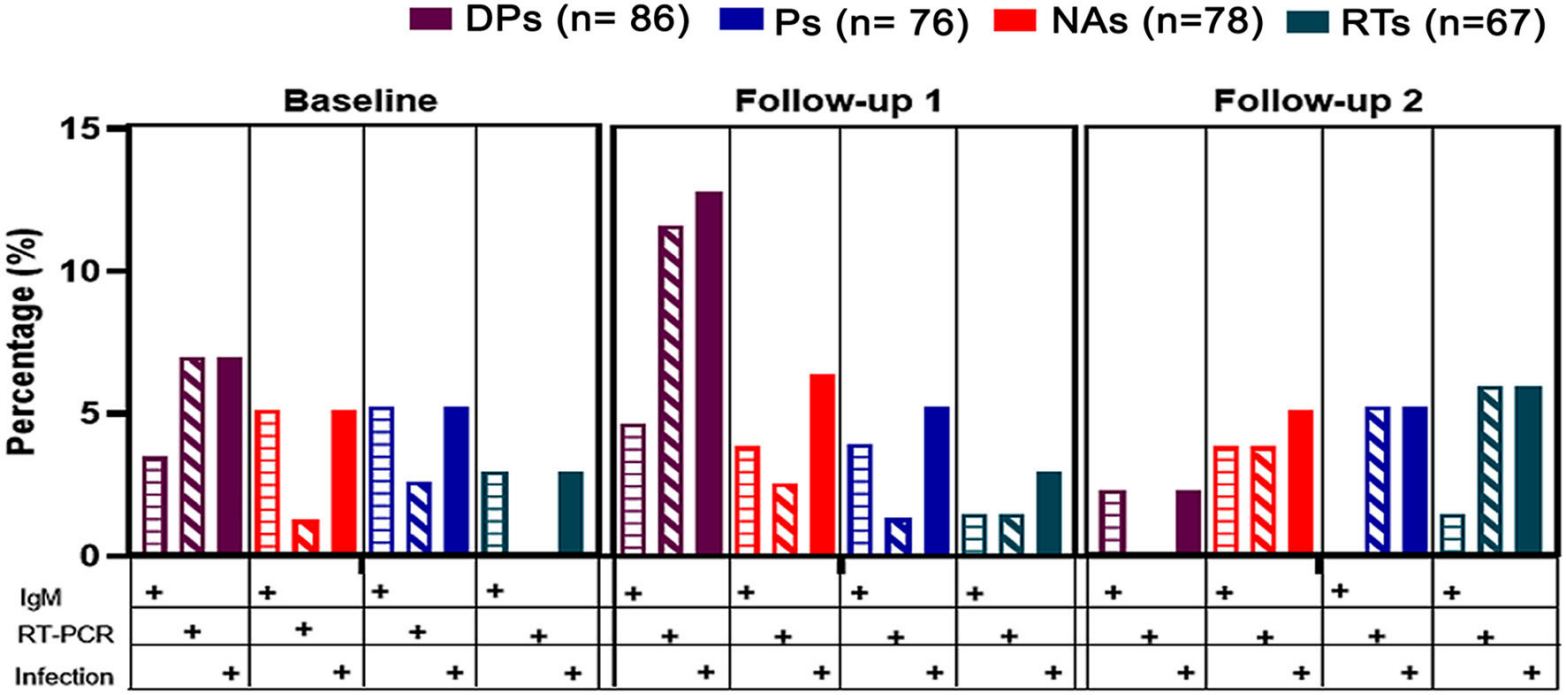
SARS-CoV-2/COVID-19 positive cases in HCWs. Cases were defined either by IgM positive result by ELFA or the amplification of the viral genome in the RT-PCR assay, in serum samples, or nasopharyngeal swabs, respectively. Colors represent the groups of HCWs with respect to the whole cohort (DPs: dental practitioners; NAs: Nurse assistants; Ps: Physicians; RTs: Respiratory therapists). No differences were found between groups (Analysis of variance (ANOVA). Index values are represented in BAU (Binding Antigen Units) per milliliter. Single plots represent the index values of antibodies in one HCW. A cutoff of 1 BAU/mL was considered to be a positive sample. Colors represent the groups of HCWs. No differences were found between groups (Analysis of variance (ANOVA).

**Figure 3:**
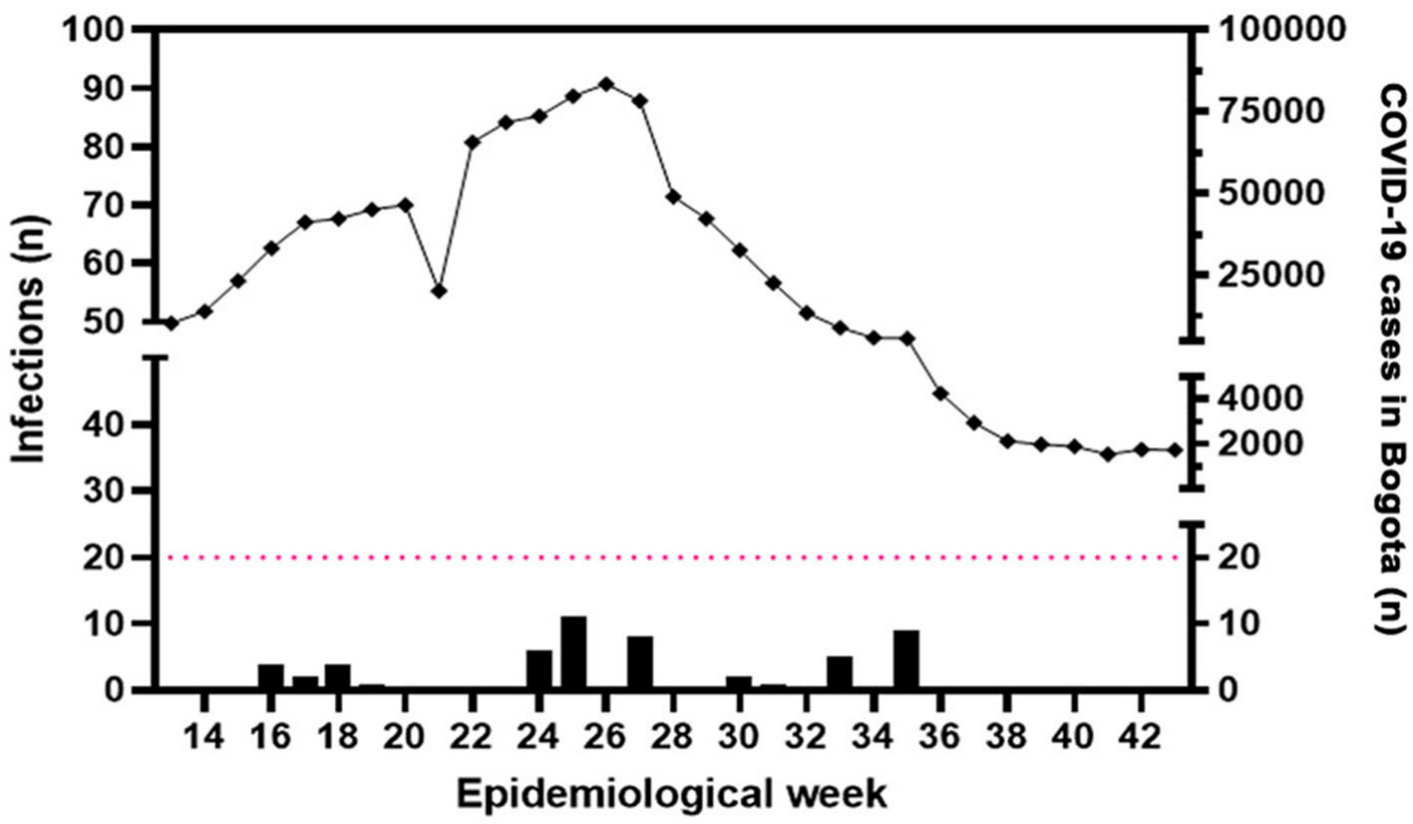
Cases of SARS-CoV-2/COVID-19 positive between HCWs. Bars represent the number of IgM and/or RT-PCR positive samples distributed during the timeline of the study. Diamonds represent the number of cases reported in Bogotá in each epidemiological week (source: https://www.paho.org/es/reportes-situacion-covid-19-colombia (23). Note the coincidence between the epidemiological peaks and the increasing number of cases in the cohort.

At BL, 5.2% (*n* = 16) of HCWs tested positive for SARS-CoV-2 in the RT-PCR test or had a positive IgM antibody result. Compared to that at BL, a higher number of HCWs (*n* = 22; 7.2%) tested positive at follow-up 1 and a lower number (*n* = 14; 4.6%) at follow-up 2 (Figures 2, 3). Among those who had tested positive for SARS-CoV-2 infection at BL, 12.5% reported not being provided with N95 respirators, and 21.4% reported not being provided a daily replacement. At follow-up 1, all those who had tested positive reported being provided with N95 respirators, but 2 (13.6%) HCWs reported not being provided a daily replacement. At follow-up 2, all those who had tested positive had been provided with N95 respirators and a daily replacement.

HCWs who tested positive for SARS-CoV-2 infection at BL did not report any symptoms, whereas the 1% who tested negative reported fever, respiratory distress, and respiratory difficulty during the 15 days preceding sample collection. Of the 16 HCWs who tested positive, one had not received any vaccine dose.

At follow-up 1, 17 HCWs reported symptoms of respiratory distress (91.67%) or respiratory difficulty (29.1%)—two HCWs (9.52%) who tested positive reported fever as the only symptom. At follow-up 2, only 1 (4.6%) HCW presented with a fever. None of the HCWs reported potential exposure to the SARS-CoV-2 virus outside the workplace.

### Exposure determinants

At BL, 17 (5.5%) HCWs reported close contact with a person diagnosed with COVID-19 at work, of whom one tested positive. Nine HCWs (2.93%) reported travels not associated with the workplace (none tested positive). Forty HCWs (13.03%) reported potential exposure to the SARS-CoV-2 outside of the workplace (social meetings, attendance in crowded indoor spaces, or tourism) 14 days prior to each sample collection, of which two tested positive (5.0%).

At follow-up 1, one (4.6%) infected HCW reported close contact with a person diagnosed with COVID-19 at work. No infections were found among participants who traveled. In addition, of the HCWs who were exposed to SARS-CoV-2 outside of the workplace, 2 (9.1%) were infected, whereas 18 (6.32%) were not. At follow-up 2, of 14 (4.5%) infected HCWs, none reported close contact with someone diagnosed with COVID-19 at work. No associated work travel or outside exposure to SARS-CoV-2 was reported.

No correlation was found between the IgG-positive status of the HCWs and their susceptibility to a new SARS-CoV-2 infection (*p* > 0.05).

## Discussion

This cross-sectional study assessed the seroprevalence of IgG antibodies, SARS-CoV-2 infection, work exposure, and PPE use in Colombian HCWs at seven primary-care-provider centers and one dental school during the COVID-19 pandemic (April–October 2021). A relatively low prevalence of active SARS-CoV-2 infection was found in the study population, with DPs showing the highest number of infections among the HCWs, at least at two of the three time points assessed. A high percentage of HCWs positive for IgG antibodies was found, indicating previous exposure to the virus or vaccine response), while IgG index values decreased during the timeline. In addition, restrictions on the use of PPE and a lack of knowledge in the current national healthcare system were identified.

HCW groups and variables analyzed and reported in this study have not been published in the Latin American region so far. The present study evidences a real-life scenario during a critical period of time when HCWs faced patient health care.

In order to have a representative sample, participants were recruited from different municipalities in the city, considering that Bogotá has the largest number of HCWs in the country. It allowed us to get an adequate approximation and a wider point of view of the situation. Based on the calculated sample size, comparisons between groups allowed for the identification of differences among them. A relevant aspect to consider regarding the data reported here is that, in Bogotá, a high proportion of HCWs are employed at more than one institution. These professionals have to commute long distances by public transport between workplaces and go home; some also used to live in higher-risk locations. Consequently, the results could be influenced by factors modifying the exposition other than those presented at their workplaces. In addition, some participants had comorbid conditions or were on concurrent medications that could have impacted their infection's susceptibility or antibody response.

In this study, regarding PPE use, the HCWs reported limited replacement of N95 masks, pointing toward a situation experienced in other countries, where the chain supply of PPE was restricted due to its time-limited availability and high cost (12). This fact did not agree with the guidelines from the National Ministry of Health in Colombia (MHC), where the use of this PPE was stated as the essential infection-control measure that must have been available for HCWs (19). Regarding the understanding of infection risk understanding, the HCWs had a split perception. Some of the HCWs percevied visiting supermarkets as a higher risk than their workplaces, while others perceived high susceptibility to SARS-CoV-2 infection in their work settings. The perception of virus exposure at the workplace could be related to the unfamiliarity of some participants with PPE or the lack of appropriate training regarding its use at the beginning of the study. In this regard, Le et al. found that HCWs perceived a high risk of being infected with SARS-CoV-2 associated with the inadequate response to COVID-19 in their own workplaces (23). In agreement with this perception, Cagetti et al. reported that a small number of dentists were confident of avoiding infection, and the level of awareness was split depending on the region of participants in Italy. This situation was mainly associated with the feasibility of adherence to national and internationally accepted guidelines (17).

Results evidenced in the current work showed a low SARS-CoV-2 infection percentage. Detected infections fit into the epidemiologic period where the highest number of SARS-CoV-2 cases were reported in Bogotá (Figure 3) (24). DPs displayed a higher number of tested positive samples in the first and second sample collections. Considering that health primary services in Colombia have a greater number of physicians and nurse assistants than dental practitioners, occupational exposure for this group would increase their susceptibility in terms of exposure and the number of patients attended by professionals. RTs had the highest positivity of infections only at the last assessment, where the vaccination status could have made them feel confident (13). Physicians and nursing assistants had a similar number of infections during the assessments; this could be related to the fact that they did not spend time at the bedside of critically ill patients or those in intensive care units, as previously reported (25–27). Merging PCR and IgM results in this work contributed to improving infection detection, leading to the identification of active SARS-CoV-2 infections with a higher sensitivity in asymptomatic HCWs.

Here, we report an increasing percentage of participants who experienced IgG seroconversion. In contrast, decreasing IgG index values were detected in serum along the timeline. Other studies have found a lower percentage of seroconversion (28). The mentioned study did not report antibody titer dynamics across time (28). They found an overall seroconversion rate of 37.0% (111/300 participants), considering positive IgG, IgM index values, or both. In the follow-up, 43% (34/79 participants) with positive index values of antibodies became seronegative (18).

However, Shields et al. found a seroprevalence of SARS-CoV-2 antibodies at BL of 16.3%. Seropositivity was retained in over 70% of participants in the next 6 months of follow-up (29). Considering the nature of the immune response, IgG SARS-CoV-2 is commonly used as an indicator of past infection and/or vaccine exposure and protection against new infections against the pathogen (30). Taken together, these issues suggest that vaccination or previous exposure to SARS-CoV-2 is not a protective factor for acquiring new infections and that symptoms are not associated with infections among HCWs.

In this study, PPE and infection were not associated. This is in agreement with findings from other authors, who show no clear association between the use of various types of PPE and the presence of infection (31–34). In addition, we found symptoms and negative laboratory tests suggesting infection with another viral agent. This finding highlights the need for public health measures to increase biosafety and limit the spread of SARS-CoV-2 and other respiratory viruses in healthcare institutions. Previous studies have highlighted the importance of wearing PPE and emphasized the use of facemasks or surgical masks to prevent the inhalation of large droplets and sprays (31–35). In this regard, the WHO recommended wearing masks as part of a comprehensive approach to reducing the spread of SARS-CoV-2 and other reports (32). In addition, education regarding the donning and doffing of PPEs should be considered. Unfortunately, this study could not directly estimate occupational exposure to the virus due to the re-activation of healthcare services, thus rendering the participants exposed to persons with positive COVID-19 status outside the workplace (31–35). Therefore, exposure to SARS-CoV-2 was explored via a survey that included reporting out-of-work exposure at social meetings or during travels.

Even though the number of participants between groups was different in the current approach, a clear trend of viral exposure was observed (DPs in BL and follow-up 1 and RT in follow-up 2). Taken together, the data obtained from this study suggests that future studies should continue merging different approaches (RT-PCR, IgM/IgG, surveys) in larger samples of HCW, and when infections are detected, a complete follow-up is suggested at different times of the infection course. These assessments might contribute to monitoring not only infections associated with SARS-CoV-2 but also the circulation of other respiratory viruses based on the current post-pandemic situation. The aspects mentioned above could contribute to a more specific characterization of the virus exposure of HCWs through cross-sectional studies and even improve existing diagnostic tests. Furthermore, more specialized studies could emphasize the long-term immune response related to vaccination and/or infection. Finally, genomic analysis could identify multiple lineages and polymorphisms in circulating SARS-CoV-2 (or other viruses).

The results of this study highlight the importance of raising awareness among HCWs regarding the risk of SARS-CoV-2 and other viral infections in the workplace. Infections in HCWs occur not only through patient care but also through exposure to close contact with colleagues (35). Public health recommendations should be strictly followed in both clinical and non-clinical areas to enhance vaccination for the entire population.

## Conclusion

Within the study's limitations, it showed a low prevalence of active SARS-CoV-2 infections by occupation, with an increasing percentage of participants with IgG seroconversion and decreasing index values of these antibodies. Further assessments could evaluate the infection risk, including clinical and non-clinical staff and their perceptions, to ensure proper training and the implementation of appropriate prevention practices that go hand in hand with the risk of exposure of each health care worker in regular care, during pandemics and non-pandemic times.

## Data availability statement

The raw data supporting the conclusions of this article will be made available by the authors, without undue reservation.

## Ethics statement

The studies involving humans were approved by Institutional Ethics Committees of Universidad El Bosque (Resolution 013, 2020) and Hospital Militar Nueva Granada (Resolution 087, 2021). The studies were conducted in accordance with the local legislation and institutional requirements. The participants provided their written informed consent to participate in this study.

## Author contributions

EB made substantial contributions to the conception and design of the work, acquisition, analysis, and interpretation of data, drafted and critically revised the manuscript, and provided the final approval of the manuscript. CC-R provided an analysis and interpreted the data, drafted and critically revised the manuscript, and provided the final approval of the manuscript. SM made substantial contributions to the conception and design of the research, as well as interpreted and analyzed data, drafted and critically revised the manuscript, and provided the final approval of the manuscript. MV-R helped with the conception and the design of the research, interpretation, analysis of data, critically revised the manuscript, and provided the final approval of the manuscript. CR-S helped with sample recruitment, collection of data, interpretation, and analysis of data, critically revised the manuscript, and provided the final approval of the manuscript. VA helped with the conception and the design of the work, analysis of data, and critically revised the manuscript and provided the final approval of the manuscript. JC made substantial contributions to the conception and design of the work, interpretation, and analysis of data, drafted and critically revised the manuscript, and provided the final approval of the manuscript. All authors contributed to the article and approved the submitted version.
